# A Novel Online Approach for Drift Covariance Estimation of Odometries Used in Intelligent Vehicle Localization †

**DOI:** 10.3390/s19235178

**Published:** 2019-11-26

**Authors:** Mostafa Osman, Ahmed Hussein, Abdulla Al-Kaff, Fernando García, Dongpu Cao

**Affiliations:** 1 Mechanical and Mechatronics Engineering, University of Waterloo, Waterloo, ON N2L 3G1, Canada; 2 Intelligent Driving Function Department, IAV GmbH, 10587 Berlin, Germany; ahmed.hussein@ieee.org; 3 Intelligent Systems Lab (LSI), Universidad Carlos III de Madrid (UC3M), 28911 Leganes, Spain; akaff@ing.uc3m.es (A.A.-K.); fegarcia@ing.uc3m.es (F.G.); 4 Waterloo Cognitive Autonomous Driving (CogDrive) Lab, University of Waterloo, Waterloo, ON N2L 3G1, Canada; dongpu.cao@uwaterloo.ca

**Keywords:** intelligent vehicles, localization, odometries drift errors, covariance estimation, adaptive filtering, ros-based

## Abstract

Localization is the fundamental problem of intelligent vehicles. For a vehicle to autonomously operate, it first needs to locate itself in the environment. A lot of different odometries (visual, inertial, wheel encoders) have been introduced through the past few years for autonomous vehicle localization. However, such odometries suffers from drift due to their reliance on integration of sensor measurements. In this paper, the drift error in an odometry is modeled and a Drift Covariance Estimation (DCE) algorithm is introduced. The DCE algorithm estimates the covariance of an odometry using the readings of another on-board sensor which does not suffer from drift. To validate the proposed algorithm, several real-world experiments in different conditions as well as sequences from Oxford RobotCar Dataset and EU long-term driving dataset are used. The effect of the covariance estimation on three different fusion-based localization algorithms (EKF, UKF and EH-infinity) is studied in comparison with the use of constant covariance, which were calculated based on the true variance of the sensors being used. The obtained results show the efficacy of the estimation algorithm compared to constant covariances in terms of improving the accuracy of localization.

## 1. Introduction

Over the past few years, the focus of several research entities as well as many industrial companies on building fully autonomous vehicles has increased drastically. One of the main aspects of building such vehicles is the localization problem, i.e., how the vehicle can determine its position relative to its environment. To address this aspect, the use of highly accurate sensors is needed such as LiDARs or differential global positioning systems. However, the use of such sensors can be very costly. Another solution is to use different sensors of lower accuracy and then apply a sensor fusion technique to the readings of all sensors in order to get a better estimate of the vehicle’s pose (position and orientation) using multiple low cost sensors [1,2,3,4,5,6].

Multisensor fusion localization can be divided into two techniques; filtering techniques, and smoothing techniques. Filtering techniques are probabilistic techniques which stem from the Bayesian estimation theory [7] and can be divided into two major directions. On one hand, there are the Gaussian filters family, and among them are the nonlinear Kalman filters such as the Extended Kalman Filter (EKF) [8], Unscented Kalman Filter (UKF) [9] and Ensemble Kalman Filter (EnKF) [10] as well as the Extended Information Filter (EIF) and the Iterative Sparse Extended Information Filter (ESIF) [11]. On the other hand, there are the nonparametric family and on top of them is the particle filter (PF) and its different versions [12].

As for smoothing techniques, these are optimization-based techniques which emerges from the need to maximize the posterior probability distribution of the vehicles pose. In other words, smoothing techniques is a Maximum a Posteriori (MAP) estimation method which comes in the form of minimizing the negative log-posterior of the vehicle pose distribution as discussed in [13]. Among such techniques are the Moving Horizon Estimation [14] as well as the factor graph method which is widely used in Simultaneous Localization and Mapping algorithms such as [15,16].

In both cases, to be able to accurately estimate the vehicle pose, the amount of noise in each of the sensors needs to be known. In almost all the cases mentioned above, the amount of noise is quantified using the covariance matrix. Either in its direct form as in the Kalman filter, as the importance factor in the particle filter as mentioned in [7], or as the information matrix in case of the information filter or the smoothing techniques.

Unfortunately, the determination of such covariance matrices is not straightforward and in most cases infeasible. To determine the covariance matrix of a sensor, the ground truth needs to be available which is generally very difficult and costly to be acquired. Furthermore, even if the ground truth is available, the localization of a vehicle in most cases depends on odometries (wheel odometry, visual odometry, inertial odometry, and so on). Such odometries relies on integration to provide the localization measurement and therefore, tend to suffer from accumulation of error. This accumulation of error has a random nature and its amount depends on the environmental conditions as well as the nature of the sensor. This in turn results in the increase of the covariance values during operation [17,18].

In this paper, we propose an online algorithm for estimating the covariance of such odometries using another on-board sensor which does not suffer from drift and with known covariance. Such a sensor only suffers from random noise and is immune to accumulation of error since it does not rely on integration. The proposed algorithm updates the drift covariance estimate with each new sensor measurement. The fact that the proposed DCE algorithm is online facilitate the estimation of the changing covariance of the odometries instantaneously during the operation of the autonomous vehicle. This makes the use of such algorithm convenient since no pre-tuning of the covariances is required.

This paper extends on the authors’ published work in [19]. In [19], the authors introduced the drift error model for wheel encoders and showed the feasibility of using an exteroceptive sensor to determine the covariance of a proprioceptive sensor such as wheel encoders. In this paper, the authors generalize the methodology and the ideas adopted in [19] to any odometry regardless of the type of sensor being used. The drift error model for any arbitrary odometry and the drift covariance formula are more rigorously derived. Furthermore, the estimation methodology introduced in [19] is generalized and more formally presented as the DCE algorithm. The generalized estimation methodology can work with any localization or SLAM techniques irrespective of its theory of action. To validate the derived error model and the proposed algorithm, a comparative study is conducted using multiple real-world experiments in different conditions as well as sequences from Oxford RobotCar Dataset [20]. To further show the benefit of using the DCE algorithm alongside with popular open source odometry algorithms, we use the open source EU long-term dataset [21] along with the LiDAR Odometry and Mapping algorithm (LOAM) [22]. We report the results of the DCE-UKF compared with the results of LOAM algorithm.

This paper’s main contributions are the following:

- The mathematical modeling of the random accumulation of error in the different odometries used in autonomous vehicle localization.
- A general online algorithmic methodology for estimating the covariance of such drift suffering odometries using another sensor which is drift-free. This methodology is generic and suits any kind of odometry as well as any localization or SLAM algorithm either being filtering-based or smoothing-based.
- The detailed implementation of an algorithm which follows this algorithmic methodology, the algorithm is available online for the benefit of the community.

To the authors knowledge, there is no previous work in the literature which addresses the problem of the covariance estimation of drift suffering odometries. Although several previous works attempt to reduce the drift in such odometries (as mentioned in Section 2), they still always suffer from drift due to their reliance on integration. The proposed work can be integrated with such drift reduction methods in order to estimate the covariance and consequently, increase the accuracy of the localization module. To show the benefit of integrating the DCE algorithm with odometries which suffer from drift, we use the LOAM algorithm alongside with the DCE-UKF and show the effect of using the DCE algorithm on the accuracy of the pose estimation compared to only relying on the LOAM algorithm for localization.

The remainder of the paper is organized as follows; Section 2 reviews the related work followed by the drift error modeling in Section 3. Section 4 describes the proposed algorithm as well as some implementation details. Then, in Section 5 the experimental setup, the localization algorithms, selected scenarios including the Oxford RobotCar Dataset and EU long-term dataset sequences as well as the evaluation metrics used for validating the algorithm are introduced. Section 6 shows the experimental results and the discussion and finally in Section 7 the conclusion is summarized.

## 2. Related Work

Ever since Rudolf Kalman proposed the Kalman filter in 1960 [23] and the continuous Kalman and Bucy filter in 1961 [24], the search for the covariances of the sensors being used in the filtering started [25]. Quantifying the noise covariance for a sensor in a multisensor fusion scheme has been the subject of several works in the literature. The difficulty in quantifying such covariances resulted in the emergence of adaptive filtering techniques [26,27].

In [28], a combination of a fuzzy logic controller and a conventional Kalman filter for an INS/GPS is proposed for the correction of both the process noise covariance and the measurement noise covariance. The algorithm was validated using a simulation with an EKF, UKF and an Iterated EKF. However, in using such algorithm, the design of the membership function for each sensor to estimate the covariance might be challenging. Especially since each sensor differs in nature and can suffer from errors in a different way. Furthermore, in case of odometries, the behavior of drift is unpredictable and designing a membership function to describe it is impractical.

Other works use adaptive Kalman filters to estimate the covariance matrices [29]. In [30], a Kalman filter with recursive estimation has been presented; to estimate the noise covariance matrix from the measurement sequence of linear time-invariant systems. Additionally, in [31], a stability analysis has been performed to verify the estimator’s stability. However, this work was introduced to linear systems and assuming that the sensors are not suffering from drift.

Beside the adaptive filtering approach which deals with the covariance of a Gaussian white noises, the fact that some sensors suffers from systematic drift errors due to aging and mis-calibration was also studied. In [32], a blind calibration algorithm is proposed to calibrate the sensor drift using signal space projection and Kalman filtering. However, in such case, the drift being addressed is the systematic drift which can be eliminated through calibration. Also, in [33], a deep learning approach is proposed to address the same issue.

In [34,35], a method based on Bayesian Maximum Entropy and Interacting Multiple Model is proposed to detect and correct the drift in sensors used in Internet of things (IoT) technologies. However, still in this case the drift error being addressed is the systematic drift of the sensors over time due to temperature conditions, vibrations and aging which can be calibrated and eliminated.

In [36,37], an online method to quantify and eliminate the random drift error in a Fiber optic gyro (FOG) is proposed through adaptive Kalman filtering. Such works only addresses the random drift noise in FOG and not in general odometries or general sensors suffering from random drift.

Most of the literature deals with drift as a systematic error which occurs due to the loss of calibration, temperature conditions, aging of the sensors or other environmental conditions. In such cases, this kind of error can be eliminated through calibration or compensation [38,39,40].

However, an odometry uses a sensor in which its measurements is being integrated over time to obtain the vehicle’s pose measurements. For example, dead-reckoning (wheel odometry) is done using a wheel encoder by integrating the output of the encoder through the kinematic model to get the pose of the vehicle. In such cases, the errors in the encoders are accumulated in the calculated pose. Another example can be a visual odometry algorithm which integrates the incremental motion of the vehicle between frames to calculate the overall pose of the vehicle.

There are several works in the literature which deals with drift in odometry. However, in these works, the methodology adopted by the authors was mainly based on reducing the error in the odometries. In [41], a visual odometry algorithm which uses a newly developed feature descriptor is introduced to reduce the drift in such visual odometry. Similarly, in [42], a convolutional neural network was used to infer the sun direction which in turn is used to reduce the drift error in the visual odometry.

In [43], a fusion scheme of monocular vision and radio-based ranging is introduced to reduce the drift error in a SLAM algorithm. In [44], a learning approach was used to solve for the drift of a LiDAR-only motion estimation. In all these works, dealing with drift was through trying to reduce it; however, drift cannot be completely eliminated, and it would be significantly useful to quantify the amount of drift in a given odometry during operation.

As already mentioned, such odometries uses sensors which suffers from various types of errors due to ground conditions (slippage), temperature changes, driving behavior or lighting conditions. As a results of integration, the error in the pose calculation will accumulate while the vehicle is operating. Furthermore, due to the change of operational conditions, the drift itself cannot be predicted and the rate at which error accumulates in the measurements of an odometry will change with time.

## 3. Drift Modeling in Odometries

A good start to model the drift in an odometry is through a proper definition of an odometry as well as drift error in odometry as seen throughout this paper.

*Definition (Odometry):* Odometry is measuring a vehicle’s pose $x\in R^{n}$ or a subset of it $z\in R^{m}$ through integrating the readings of a sensor after mapping it to the state (output) space,

(1) $$\underset{z_{k}}{\underset{︸}{{\mathrm{Cx}}_{k}}}=\underset{z_{k-1}}{\underset{︸}{{\mathrm{Cx}}_{k-1}}}+\underset{\Delta z_{k}}{\underset{︸}{g(q)}},$$

where $C\in R^{m\times n}$ is the binary output matrix and for $C=I_{n\times n}$, the odometry measures the overall state vector x, $q\in R^{p}$ is the raw measurement of the sensor used in the odometry, $g:R^{p}\to R^{m}$ is the odometry mapping (maps the sensor measurements to the odometry output increments), and k∈N is the timestep where t=kT and $T\in R_{\geq 0}$ is the sampling time and $t\in R_{\geq 0}$ is the time in seconds.

Notice that an odometry measures the position of a vehicle or a robot, consequently, the state vector can be defined as

(2) $$x:={\left[\begin{array}{cccccc} x & y & z & \alpha & \beta & \gamma \end{array}\right]}^{T},$$

where *x*, *y* and *z* are the three Cartesian coordinates of the vehicle and α,β and γ are pitch, roll and yaw angles of the vehicle, respectively. Some odometries also provide the accelerations or the velocities, in such cases, these states can also be added to the state vector.

*Definition (Drift Error):* The drift error $\delta \left(z,k\right)\in R^{m}$ is the unbounded accumulation of error in the odometry measurements due to the integration of the noisy readings, where for the drift error, the following is true.

(3a) $$\delta \left(0_{m\times 1},0\right)=0_{m\times 1},$$

(3b) $$\begin{array}{ccccc} \lim_{z\to \infty }\delta \left(z,\cdot \right)=\infty , & \; & \mathrm{and} & \; & \lim_{k\to \infty }\delta \left(\cdot ,k\right)=\infty . \end{array}$$

Now consider the raw measurement of a sensor q used in an odometry, where the readings of the sensor is related to the states of the vehicle through the sensor model mapping $h:R^{n}\to R^{p}$.

(4) $$q_{k}=h\left(x_{k}\right)$$

Equation (4) can be written more precisely as a function of the observable states by the sensor $h:R^{m}\to R^{m}$ as follows,

(5) $$q_{k}=h\left({\mathrm{Cx}}_{k}\right)=h\left(z_{k}\right),$$

since the measurements of the sensor is only a function of the related states which in this case is the z output vector.

Consequently, the ideal odometry equation will be

(6) $$z_{k}=z_{k-1}+g\left(q_{k}\right)=z_{k-1}+g\left(h\left(z_{k}\right)\right).$$

Although the sensor measurement $h(z_{k})$ is a function of the output vector $z_{k}$, this is an implicit representation and the only way to measure the actual output vector $z_{k}$ is through the odometry.

In reality, the readings of the sensors q suffers from noises which can be represented as

(7) $$\hat{q}=h\left(z\right)+\epsilon ,$$

where $\hat{q}$ is the noisy readings of the sensors and $\epsilon \in R^{p}$ is a random variable representing the additive noise on the sensors.

Using Equation (7) in (6), the noisy odometry output can be written as follows:

(8) $${\hat{z}}_{k}={\hat{z}}_{k-1}+g\left(q_{k}+\epsilon_{k}\right)$$

Using the Taylor expansion assuming that $g\circ h_{q}$ is a $C^{\infty }$ smooth function, Equation (8) can be expanded as follows:

(9) $${\hat{z}}_{k}={\hat{z}}_{k-1}+g\circ h\left(z_{k}\right)+\frac{\partial g}{\partial q_{k}}\epsilon_{k}+\epsilon_{k}^{T}\frac{\partial^{2}g}{\partial q_{k}^{2}}\epsilon_{k-1}+\cdots ,$$

where

$$\begin{array}{ccc} \frac{\partial g}{\partial q_{k}}=:J_{k}\in R^{m\times n}, & \; & \frac{\partial^{2}g}{\partial q_{k}^{2}}=:H_{k}\in R^{n\times m}, \end{array}$$

are the Jacobian matrix and the Hessian matrix and so on.

Equation (9) can also be rewritten as follows:

(10) $${\hat{z}}_{k}=z_{k}+\underset{\delta (z,\epsilon ,k)}{\underset{︸}{\sum_{i=0}^{k}J_{i}\epsilon_{i}+\epsilon_{i}^{T}H_{i}\epsilon_{i}+\cdots }},$$

where $\delta \in R^{m}$ is the random drift error in the odometry. The reason that the drift is random is the fact that it depends on the random noise in the sensor measurement ϵ.

Now assume a change Δz occurred in the pose of the vehicle over a one timestep. Once again, through using the Taylor expansion, the drift error can then be written as follows:

(11) $$\delta \left(z+\Delta z,\epsilon ,k\right)=\delta \left(z,\epsilon ,k-1\right)+\frac{\partial \delta }{\partial z}\Delta z+\Delta z^{T}\frac{\partial^{2}\delta }{\partial z^{2}}\Delta z+\cdots$$

and the increment of the random drift error Δδ can be written as:

(12) $$\Delta \delta =\underset{K_{1}}{\underset{︸}{\frac{\partial \delta }{\partial z}}}\Delta z+\Delta z^{T}\underset{K_{2}}{\underset{︸}{{\frac{\partial^{2}\delta }{\partial z}}_{2}}}\Delta z+\cdots$$

where $K_{1}\in R^{m\times m}$ and $K_{2}\in R^{m\times m}$ are the so-called drift coefficient matrices and are random matrices which depend on the sensor random noise ϵ. This in turn makes Δδ and δ random variables.

*Assumption:* Since normally the sampling time *T* is small, Δz is small and the higher order terms can be neglected.

(13) $$\Delta \delta \approx K_{1}\Delta z$$

Furthermore, we assume that the drift in each of the output states is independent from other states, in other words,

(14) $$K_{1}=diag\left\{k_{11},k_{22},\cdots ,k_{mm}\right\}.$$

Using Equation (13) in (11), the drift error at timestep *k* can then be written as:

(15) $$\delta_{k}=\delta_{k-1}+\Delta \delta_{k}\approx \delta_{k-1}+K_{1}\Delta z$$

The covariance of the drift increment $Q_{\delta }\in R^{m\times m}$ can then be derived through the definition of covariance as follows:

$$\begin{array}{cc} Q_{\delta } & :=E[{\left(K_{1}\Delta z-\mu_{\delta }\right)}^{T}\left(K_{1}\Delta z-\mu_{\delta }\right)]\\ \; & =E[\Delta z^{T}K_{1}^{T}K_{1}\Delta z-2\mu_{\delta }^{T}K_{1}\Delta z+\mu_{\delta }^{T}\mu_{\delta }]\\ \; & =\Delta z^{T}E\left[K_{1}^{T}K_{1}\right]\Delta z-2\mu_{\delta }^{T}E\left[K_{1}\Delta z\right]+\mu_{\delta }^{T}\mu_{\delta }\\ \; & =\Delta z^{T}E\left[K_{1}^{T}K_{1}\right]\Delta z-\mu_{\delta }^{T}\mu_{\delta }, \end{array}$$

where $\mu_{\delta }:=\mu_{k}\Delta z\in R^{m}$ is the mean (first moment) of the drift increment and $\mu_{k}\in R^{m\times m}$ is the mean of the drift coefficient.

*Assumption:* The drift increment is a small value due to the small timestep and therefore the square of its mean $\mu_{\delta }$ is also small and can be neglected. Therefore, the drift covariance $Q_{\delta }$ can be written as follows:

(16) $$Q_{\delta_{k}}=\Delta z^{T}\underset{Q}{\underset{︸}{E[K_{1}^{T}K_{1}]}}\Delta z,$$

where $Q\in R^{m\times m}$ is the drift coefficient covariance matrix.

Using Equation (13), the drift covariance matrix $\Sigma_{\delta }\in R^{m\times m}$ can be calculated as

(17) $$\Sigma_{\delta_{k}}=\Sigma_{\delta_{k-1}}+Q_{\delta_{k}}=\Sigma_{\delta_{k-1}}+\Delta z^{T}Q\Delta z.$$

Using the drift model stated in Equation (17), the drift covariance matrix $\Sigma_{\delta_{k}}$ can be estimated using a sensor which does not suffer from drift as will be shown in the following section.

## 4. Drift Covariance Estimation

Using the model developed in the previous section, the drift covariance $\Sigma_{\delta }$ can be estimated using a sensor which does not suffer from drift. Throughout the rest of the paper, we call a sensor not suffering from sensor a drift-less sensor.

A good example for a drift-less sensor is a GPS which does not rely on integration for measuring the pose of the vehicle. On the other hand, an odometry can be an encoders odometry or a visual odometry. Other examples of drift-less sensors could be a camera for detecting landmarks or apriltags, a magnetometer, and so on.

### 4.1. Drift Covariance Estimation Algorithm

To avoid ambiguity, from now on, a measurement from an odometry will be denoted ${\hat{z}}_{\delta_{k}}$ and a drift-less sensor measurement will be denoted ${\hat{z}}_{k}$.

Now consider a drift-less sensor measurement ${\hat{z}}_{k}\in R^{m}$ with a known covariance $R_{k}\in R^{m\times m}$. The true output of the vehicle $z_{k}$ most likely lies in the confidence ellipse of the measurement defined by the eigenvalues of R. Since the true output $z_{k}$ is not available. We resort to sampling the measurement distribution $p({\hat{z}}_{k}x_{k})$ deterministically.

(18) $$Z_{k}:=\left[\begin{array}{ccccc} {\bar{z}}_{0_{k}} & {\bar{z}}_{1_{k}} & {\bar{z}}_{2_{k}} & \cdots & {\bar{z}}_{q_{k}} \end{array}\right],$$

where $Z_{k}\in R^{m\times q}$, and *q* is the number of samples taken from the drift-less measurement distribution, and ${\bar{z}}_{i_{k}}$ is the *i*th sample in $Z_{k}$ and ${\bar{z}}_{0_{k}}={\hat{z}}_{k}$.

Using the samples in $Z_{k}$, the innovation between the odometry output and the samples are calculated as follows:

(19) $$v_{i_{k}}=e\left({\bar{z}}_{i_{k}},{\hat{z}}_{\delta_{k}}\right),$$

(20) $$V_{k}:=\left[\begin{array}{ccc} v_{0_{k}} & v_{1_{k}} & \cdots v_{q_{k}} \end{array}\right],$$

where $V\in R^{m\times q}$ is called the innovation matrix, and $e:R^{m}\times R^{m}\to R^{m}$ is the innovation (error) function which can be defined simply as $e\left({\bar{z}}_{i_{k}},{\hat{z}}_{\delta_{k}}\right):={\bar{z}}_{i_{k}}-{\hat{z}}_{\delta_{k}}$ in case of Cartesian states (position, linear velocity and linear acceleration), but needs more elaborate design in case of orientations to manage to calculate the innovation over the rotation manifold taking into account representation singularities. For more information, see [45,46].

The innovation values are stored in the so-called innovation memory tensor $\Xi \in R^{n_{v}\times m\times q}$ which stores the last $n_{v}$ innovation matrices as shown in Equation (21). Here, we call $n_{v}$, the *measurement horizon*. The choice of the measurement horizon $n_{v}$ is a design parameter and would be a trade-off between accuracy of the covariance estimation and the computational cost.

(21) $$\Xi ={\left[\begin{array}{cccc} \Xi_{0} & \Xi_{1} & \cdots & \Xi_{q} \end{array}\right]}^{T}={\left[\begin{array}{cccc} \left[\begin{array}{c} v_{0_{k-n_{v}}}^{T}\\ v_{0_{k-n_{v}+1}}^{T}\\ \vdots \\ v_{0_{k}}^{T} \end{array}\right] & \left[\begin{array}{c} v_{1_{k-n_{v}}}^{T}\\ v_{1_{k-n_{v}+1}}^{T}\\ \vdots \\ v_{1_{k}}^{T} \end{array}\right] & \cdots & \left[\begin{array}{c} v_{q_{k-n_{v}}}^{T}\\ v_{q_{k-n_{v}+1}}^{T}\\ \vdots \\ v_{q_{k}}^{T} \end{array}\right] \end{array}\right]}^{T},$$

where $\Xi_{i}\in R^{n_{v}\times m}$ is the matrix containing the $n_{v}$ previous innovations calculated for a given track of sample points.

As mentioned before, a drift-less sensor does not suffer from error accumulation, but only from random error. Now let us recall the drift model in the previous section.

(22) $$\begin{array}{ccccc} \Delta \delta =K_{1}\Delta z, & \; & \mathrm{and} & \; & \frac{\partial \delta }{\partial z}=K_{1}. \end{array}$$

The drift coefficient $K_{1}$ represent the increase in the drift in the output of the odometry with respect to the change in the output states z. Therefore, using a drift-less sensor, the change in the states due to drift can be captured by estimating the $K_{1}$.

After we stored the innovation points for $n_{v}$ sample points, which encodes the deviation between the odometry and the drift-less sensor. The slope of these values is an estimate of $K_{1}$ since the normal progression of the output states z is captured by both sensors but only the drift suffering sensor has the drift component δ.

In other words, using the innovation memory tensor Ξ, the drift coefficient $K_{1}$ can be estimated for each track of the sample points, by computing the first order polynomial fit of the innovation data using a linear least squares approach as shown in the following equation.

(23) $$\left[\begin{array}{c} \tau \\ {\hat{K}}_{i}{]}^{T} \end{array}\right]={\left({\bar{T}}^{T}\cdot \bar{T}\right)}^{-1}{\bar{T}}^{T}\Xi_{i},$$

(24) $$\bar{T}=\left[\begin{array}{cc} 1_{n_{v},1} & T \end{array}\right],$$

(25) $${\left[{\hat{K}}_{i}\right]}^{T}=\left[\begin{array}{ccc} {\hat{k}}_{11} & \cdots & {\hat{k}}_{1m} \end{array}\right],$$

where $T\in R^{n_{v}}$ is the time vector for the last $n_{v}$ instances, ${\hat{K}}_{i}\in R^{m\times m}$ is the estimated drift coefficient matrix computed for $\Xi_{i}$ and $\left[{\hat{K}}_{i}\right]\in R^{m}$ is a vector containing the diagonal values of the drift coefficient matrix, $1_{n_{v},1}\in R^{n_{v}}$ is an all ones vector, and $\tau \in R^{1\times m}$ is the intercept which is of no use here. Equation (23) is calculated for each sub-matrix $\Xi_{i}$ and *q* estimates of $K_{1_{k}}$ are calculated.

Using these estimates, the drift coefficient covariance Q can be also estimated using Equation (16) as

(26) $${\hat{Q}}_{k}=E\left[K_{1}^{T}K_{1}\right]=\frac{1}{q}\sum_{i=0}^{q}{\hat{K}}_{i}^{T}{\hat{K}}_{i}.$$

Consequently, the drift increment covariance and the drift covariance matrices can be calculated as

(27) $${\hat{Q}}_{\delta_{k}}=\Delta {\hat{z}}_{\delta_{k}}^{T}{\hat{Q}}_{k}\Delta {\hat{z}}_{\delta_{k}},$$

(28) $${\hat{\Sigma }}_{\delta_{k}}={\hat{\Sigma }}_{\delta_{k-1}}+W{\hat{Q}}_{\delta_{k}},$$

where $W\in R^{m\times m}$ is a diagonal matrix of weights. Notice that in Equation (27), since the true output state increment $\Delta z_{k}$ is not available, and assuming a fast sampling time, the increment from the odometry is used instead. To compensate for such change in the equation, the design parameter W is added to the algorithm. The covariance estimation algorithm is stated in Algorithm 1. 

**Algorithm 1:** Drift Covariance Estimation Algorithm.

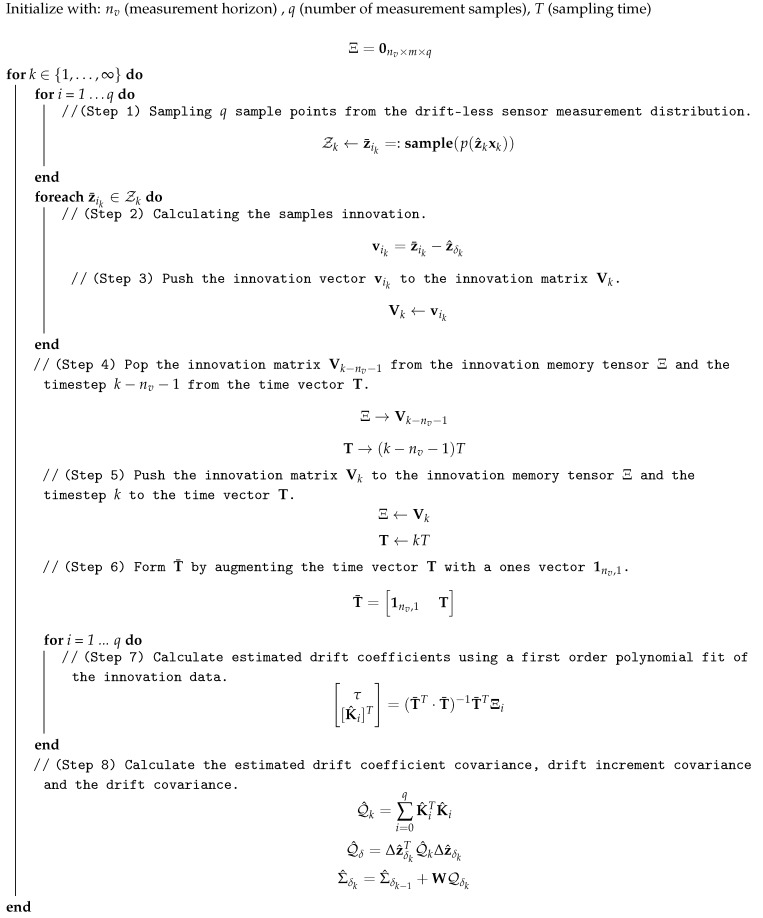



### 4.2. Algorithm Implementation

A generic implementation of the covariance estimation algorithm was developed and available as online open source repository in [47]. This implementation uses the unscented transform introduced in [48,49]. Normally, the unscented transform is used in the unscented Kalman filter; however, it also fits for the proposed covariance estimation algorithm for sampling the drift-less measurement distribution.

Sampling a drift-less measurement distribution using the unscented transform is as follows.

(29) $$Z_{k}=\left[\begin{array}{ccc} {\hat{z}}_{k} & {\hat{z}}_{k}+\left(\alpha \sqrt{R_{k}}\right) & {\hat{z}}_{k}-\left(\alpha \sqrt{R_{k}}\right) \end{array}\right]\in R^{m\times 2m+1}$$

where α∈R is the sampling parameter in the unscented transform.

The α parameter is normally calculated as α=m+λ where lambda is a design parameter in the unscented transform (for more information see [49]). This calculation method can be used for sampling the measurement distribution. However, to show that the algorithm is independent of the sampling approach, here we make the α itself a design parameter which can be set by the user without the need to specify λ first

*Remark:* The choice of the parameter α should be dependent on the accuracy of the drift-less sensor covariance. More specifically, if the drift-less sensor covariance $R_{k}$ reflects under-confidence in the sensor, then the true pose z is more likely to be in the confidence ellipse and consequently, α should be less than 1. However, if the covariance reflects over-confidence in the sensor, then α should be greater than 1.

Also, similar to the unscented Kalman filter, rather than calculating the normal mean for the drift coefficients, we calculate a weighted mean. The weights calculations and the covariance calculation are described in the following equations.

(30) $$W_{0}=\frac{m}{2m+1}\mathrm{and}W_{i}=\frac{m+1}{4m^{2}+2m},$$

(31) $${\hat{Q}}_{k}=\sum_{i=0}^{2m}W_{i}^{\left(m\right)}{\hat{K}}_{i}^{T}{\hat{K}}_{i},$$

where $W_{i}\in R$ is the weight for a given sampled measurement.

*Remark:* The choice of the weights stated in Equation (30) is a design choice. The calculation of the weights is different than that of the unscented transform. Here, we chose to give a higher weight to the sensor measurement $\hat{z}$ while giving equal weights to all other samples. Again, this is a design parameter which can be changed or even completely removed from the algorithm implementation.

**Figure 1:**
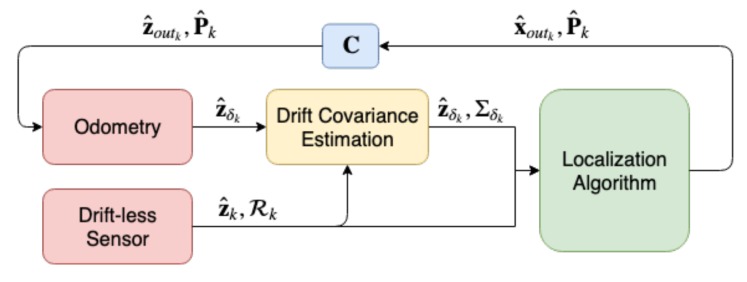
The covariance estimation algorithm operation structure with the corrective feedback localization from the localization algorithm to the odometry.

Notice that the proposed algorithm only estimates the covariance of the odometry output ${\hat{z}}_{\delta }$ but does not correct such drift. The correction can be done through using the output of the localization algorithm ${\hat{x}}_{out}$ as shown in the following equations and illustrated in Figure 1.

(32) $${\hat{z}}_{\delta_{k}}={\mathrm{Cx}}_{out_{k-1}}+\Delta {\hat{z}}_{\delta_{k}}$$

(33) $${\hat{\Sigma }}_{\delta_{k}}={\hat{P}}_{\delta_{k-1}}+W{\hat{Q}}_{\delta_{k}}$$

Through using the drift covariance estimation, over time the covariance of the odometry will be more accurately quantified taking the odometry drift into consideration. This odometry with its estimated covariance is then provided to the localization algorithm which will help conserve an accurate localization output which in turn will be fed to the odometry to correct the drift.

#### Time and Memory Complexity

In this subsection, the time and memory complexity of the algorithm described in the last subsection are presented. As can be seen in Algorithm 1, the most computationally expensive operation is the sampling of the measurement distribution due to the matrix square root operation. The matrix square root is typically performed using Cholesky decomposition and in general the time complexity would be O($m^{3}$) [50]. Although this is normally computationally expensive, in this case, the worst-case scenario covariance matrix would be a 6×6 matrix which still considered a simple operation due to the powerful processors available presently. Furthermore, if the noise in the sensor readings is uncorrelated (for different states), the covariance matrix reduces to a diagonal matrix and the matrix square root reduces to a set of normal square root operations which is a constant time operation. Consequently, ignoring the matrix square root, several steps of the algorithm iterates over each sampled measurement making the algorithm complexity O(*q*). Notice that the matrix inversion of $\left({\bar{T}}^{T}\cdot \bar{T}\right)$ is only an inversion operation of a 2×2 matrix which is a constant time operation.

As for the memory complexity, this can be specified using the innovation memory tensor. In each iteration of the algorithm, the innovation memory tensor must store the *m* measured states for each of the *q* samples generated for each of the timesteps in the measurement horizon $n_{v}$. This makes the memory complexity of the algorithm O($mqn_{v}$).

## 5. Experimental Work

To validate the proposed algorithm, several scenarios were designed and tested over various experiments as well as Oxford RobotCar Dataset [20]. Furthermore, to show the effect of the DCE algorithm on the localization results compared to open source odometries, we report the results of using an open source implementation of the LOAM algorithm along with the EU long-term dataset compared to that of using the same package alongside a DCE-UKF algorithm. This section describes the used platform for the real-world experiments, the testing environment, the designed scenarios, the multisensor fusion filters used in testing the algorithm, the selected evaluation metrics, and the sessions used for validation from the Oxford RobotCar Dataset and EU long-term dataset.

### 5.1. Experimental Platform

The aforementioned approach was tested over an automated ground vehicle, which is a part of the Intelligent Campus Automobile (iCab) project [51]. The vehicle is an electric golf cart, which is equipped with multiple on-board sensors, including LiDAR, stereo camera, optical encoders for wheel and steering, digital magnetometer and GPS module. Moreover, the vehicle is equipped with an embedded computer, which operates with Robot Operating System (ROS)-based architecture [52]. This architecture enables the vehicle to perform self-localization, navigation, planning and environment perception, among others.

### 5.2. Scenarios

**Figure 2:**
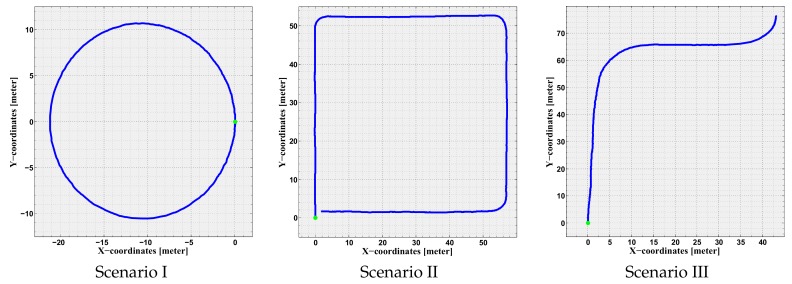
Visual demonstration of the three scenarios, where the green point is the start point and the blue curve is the path drawn using the LiDAR odometry. The Figure is reproduced from [19] (© 2018 IEEE).

The testing environment was the off-road vicinity of the campus, which has free pedestrian areas and surrounded with many buildings. In this environment, three scenarios were designed to evaluate the proposed approach, and each scenario was experimented three times under different conditions. The scenarios are depicted in Figure 2 and described in the following subsections.

#### 5.2.1. Scenario I

The first scenario was designed as a circle of total diameter of 22 m, in which the iCab steering wheel was adjusted to 8.5${}^{\circ }$ and average velocity of 5 kmph. In this case, the theoretical path was designed as a pure circle with the same diameter to be compared with the obtained odometries. The scenario was selected to evaluate the proposed approach performance in optimizing the localization in simple circular motion. Moreover, it is a closed loop, thus the vehicle end point is the same as the starting point.

#### 5.2.2. Scenario II

The second scenario was designed as a quadratic shape of total length of 54 m and width of 56 m. This was also a closed shape, where the theoretical path was designed as a right-angled quadratic shape, thus the vehicle end point is the same as the starting point. The iCab followed the shape with average velocity of 5 kmph and rotating with sharp $30^{\circ }$ around the corners. The scenario was selected to evaluate the proposed approach performance in optimizing the localization in both straight-line and curved motions.

#### 5.2.3. Scenario III

The third and last scenario was designed as a trajectory from one building to another in the campus. The selected points are part of the pick-up/drop-off points of the iCab project. The theoretical path was obtained from the path planner to be compared with the obtained odometries. The path consists of multiple curves, straight-lines, dynamic obstacles, and it is one of the normal trajectories that the iCab follows in its daily operation. The iCab followed the path with average velocity of 5 kmph and a maximum of 15${}^{\circ }$ steering angle during curvatures.

For the iCab experiments, we chose to use the velodyne sensor along with the LOAM algorithm as a reference odometry since the ground truth was absent through the experiments. The reason for choosing the LOAM algorithm (which is also considered an odometry) is the fact that package accuracy over the KITTI Benchmark [53] is in the second place in the list of the most accurate odometry algorithms, with values of 0.59% and 0.0014${}^{\circ }$/m for the $TE_{mean}$ and $OE_{mean}$ (defined in the evaluation metrics subsection) respectively. Furthermore, The iCab experiments were executed with low speeds which enhance the accuracy of the LOAM algorithm as can be seen in Figure 2. Therefore, LOAM readings were considered to be the closest to ground truth in comparison with all other available sensors in the iCab, and accordingly it was set as the reference odometry through the experiments.

### 5.3. Oxford RobotCar Dataset

The Oxford RobotCar is a dataset for autonomous driving which was recorded through the period of May 2014 till December 2015 through traversing a fixed route through central Oxford using Oxford RobotCar platform which is an Autonomous Nissan LEAF [20]. The autonomous vehicle used is equipped with 6 Cameras, a LiDAR, GPS and INS + GPS as the ground truth.

In this paper, several sessions were used from the dataset to verify the covariance estimation algorithm. In the dataset, the GPS data contains its covariance. Accordingly, a multisensor fusion algorithm can be used to fuse the GPS data with visual odometry data also provided by the dataset.

Given that the visual odometry algorithm suffers from drift, the covariance of the visual odometry is estimated using the proposed algorithm with the GPS covariance then the localization using both visual odometry and GPS measurements. Moreover, since these are pre-recorded data, no correction was made to the results of the odometry. These tests are useful to validate the covariance estimation even without correction of the odometry output and its covariance.

### 5.4. EU Long-Term Dataset

The EU long-term Dataset [21] is a dataset for autonomous driving which contains the sensors data from multiple heterogeneous sensors. The data was collected using the UTBM car in human driving mode through driving the car in the downtown of Montpelier in France. For the long-term data, the driving distance was about 5.0 km per session which was driven over 16 min.

In this paper, we use the LiDAR data to validate the DCE algorithm. Since the GPS/RTK data from the dataset is the ground truth, we induced an artificial noise over its data and used it as a drift-less sensor for the covariance estimation. Furthermore, an open source implementation of the LOAM algorithm was used as a LiDAR odometry suffering from drift. Here, because the dataset was recorded with a relatively high speed (50 kmph), the results of the LOAM algorithm deteriorated very fast and showed a considerable amount of drift. Therefore, it was suitable for comparison with the DCE-UKF results.

### 5.5. Multisensor Fusion-Based Localization Algorithms

To evaluate the true potential of the proposed algorithm, the performance and results are compared relative to different values of constant covariances in a different multisensor fusion algorithms, and the robot_localization package was selected in order to do the fusion [54]. The package contains two fusion algorithms: Extended Kalman Filter (EKF) and Unscented Kalman Filter (UKF). Also, a third filter was added by the author to the robot_localization which is the Extended H-infinity filter.

#### 5.5.1. Extended Kalman Filter

The Extended Kalman Filter(EKF) is the direct extension of the most widely used filter for sensor noise filtering which is the Kalman filter algorithm [23]. EKF uses the first order Taylor expansion to linearize the nonlinear system and propagate them using the error propagation theory. The EKF is a recursive algorithm which provides the optimal minimum mean-squared error (MMSE) state estimation assuming that the prior predictions and observations are both Gaussian random variables.

#### 5.5.2. Unscented Kalman Filter

The Unscented Kalman Filter (UKF) is another nonlinear extension of the linear Kalman filter algorithm [48,49,55]. However, in case of the UKF, the algorithm deals with the nonlinear system directly without the need for linearization which leads to increased accuracy of the state estimation. UKF uses the unscented transform to propagate the error through the nonlinear system directly but still in this case both the prior prediction and observation are assumed to be Gaussian random variables.

#### 5.5.3. Extended H-Infinity Filter

The Extended H-infinity filter is another probabilistic filter which aims at minimizing a different objective than that of the Kalman filters [56]. In EH-infinity, a game theory approach is used in which nature is considered the opponent of the filter and is trying to maximize the errors in the estimation. In other words, the EH-infinity assumes the worst-case scenario in which both the estimation and observation errors, are maximum [57]. This worst-case scenario is modeled by the cost function which is then converted to a minimax problem and the EH-infinity solves this minimax problem.

In this paper, the three filters are used to show the efficacy and accuracy of the covariance estimation algorithm being proposed. The choice of using robot_localization package was made to use an already verified and published fusion package, thus focusing on the effect of using different covariances under the same conditions.

### 5.6. Evaluation Metrics

The evaluation metrics for the sensor fusion algorithms were calculated for both translation and orientation. First, the mean and maximum error percentages of the translation; which are calculated as shown in Equations (34) and (35) respectively.

(34) $$TE_{mean}\left[\%\right]=\frac{\frac{1}{N}\sum_{k=1}^{N}{∥\left[\begin{array}{c} {\hat{x}}_{k}\\ {\hat{y}}_{k} \end{array}\right]-\left[\begin{array}{c} x_{k}\\ y_{k} \end{array}\right]∥}_{2}}{TotalDistance},$$

(35) $$TE_{max}\left[\%\right]=\frac{max({∥\left[\begin{array}{c} {\hat{x}}_{k}\\ {\hat{y}}_{k} \end{array}\right]-\left[\begin{array}{c} x_{k}\\ y_{k} \end{array}\right]∥}_{2})}{TotalDistance},$$

where $\left({\hat{x}}_{k},{\hat{y}}_{k}\right)$ are the estimated coordinates of the vehicle and $\left(x_{k},y_{k}\right)$ are the true coordinates at time step *k*.

As for the orientation, the mean and maximum error in the orientation are divided by the total distance covered by the vehicle, which are calculated as shown in Equations (36) and (37) respectively.

(36) $$OE_{mean}{[}^{o}/m]=\frac{\frac{1}{N}\sum_{k=1}^{N}{\hat{\theta }}_{k}-\theta_{k}}{TotalDistance},$$

(37) $$OE_{max}{[}^{o}/m]=\frac{max({\hat{\theta }}_{k}-\theta_{k})}{TotalDistance},$$

where ${\hat{\theta }}_{k}$ is the estimated orientation of the vehicle and $\theta_{k}$ is the true orientation of the vehicle at time step *k*.

## 6. Results and Discussion

Five different sensors were used in the iCab platform through experiments; the LiDAR as the reference odometry, GPS as a drift-less sensor, wheel encoders odometry and visual odometry [58]. Additionally, compass orientation was included to the fusion algorithm. The covariance was estimated for the odometries (Encoders and Visual) using the available covariance of the drift-less sensor (GPS). For the covariance estimation algorithm, the encoder odometry measured the three planar states of the vehicle ${\left[x,y,\gamma \right]}^{T}$ while the visual odometry only estimated the planar position of the vehicle ${\left[x,y\right]}^{T}$.

As for Oxford RobotCar Dataset, the sessions used in this paper are a total of four days driving in the dataset which is about 7165 meters driving. However, in the case of the dataset, only the translation errors are calculated due to the absence of measurements of the orientation.

Furthermore, for the EU long-term dataset, a total of three sessions were used for the comparison, these are a total of 15,000 km of driving divided over three days in different weather conditions. Similar to Oxford RobotCar Dataset, only the translational ground truth data are available in the dataset (from the GPS/RTK), so here we only show the result of the fusion for the translation.

To show the efficacy of the Drift Covariance Estimation (DCE) algorithm, its localization results are compared to the results of different values of constant covariances. The values used are percentages of the True Variances (TV) of the odometries, which are calculated retroactively from the data of the experiments.

The constant values of covariances are selected depending on the error values for each experiment. As shown in the tables below, 125% of the TV gave higher error than 25% of the TV. Observing these results, smaller percentages of the TV were used in order to reach better accuracy. In addition, lower than 2.5% of the TV were omitted, because they also provided less accurate results.

All the data from the different scenarios are fused using the three filters mentioned in Section 5. It is worth mentioning that the results from each filter are taken separately as no direct comparison between the filters is intended in the paper. For example, the EH${}_{\infty }$ filter contain a parameter called the performance bound which needs tuning and can significantly enhance the results of the filter; however, the enhancement of the filter results is out of scope since the main concern is to validate the efficacy of the DCE algorithm for different fusion algorithms using the same filter parameters for constant and adaptive covariances.

Also, notice that the correction step was used through the experiments of the iCab and the EU Long-term dataset. The covariance for the odometries based on the TV was calculated as follows:

(38) $${\hat{\Sigma }}_{k}={\hat{P}}_{k-1}+Q_{t}\;\Delta z,$$

where $Q_{t}\in R^{m\times m}$ is the covariance matrix containing the true variances for each of the output states.

### 6.1. Platform Experiments

**Figure 3:**
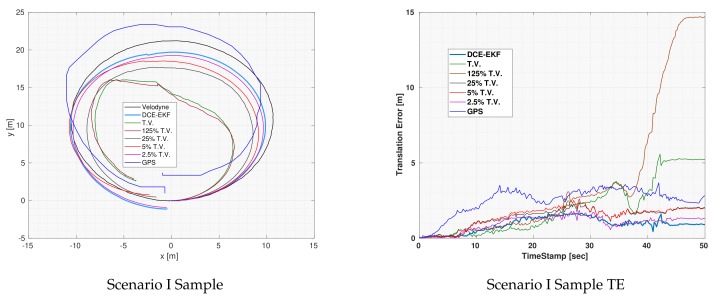
Visual demonstration of one of Scenario I experiments using EKF for localization.

In Figure 3, one of the three experiments which were executed for Scenario I using the EKF is shown as well as the error plots for each of the covariance values and the GPS which was used to estimate the covariance of the odometries. Notice that for the TV and the 125% of TV, the output of the EKF diverged which means that even the use of the true variance did not result in good fusion results. This is expected because the covariance of an odometry should increase with time as the drift error increases in the measurements. Overall, the DCE-EKF outperformed all other covariance values as can be seen in the error plot.

Notice that although the TV was outperforming the DCE algorithm during the first 25 seconds of the experiment, as shown in the translation error plot, the DCE algorithm had significantly more accurate results afterwards. This is a result of the fact that the DCE relies on previous data to estimate the covariance and at the beginning of the experiment, there was no enough data to estimate the covariance accurately and the GPS was suffering from increasing error imitating that of an odometry (as shown in Figure 3). However, after having enough data the DCE algorithm managed to converge to a much better estimate of the covariance and consequently of the vehicle’s location.

Table 1, Table 2 and Table 3 summarizes the quantitative results for Scenario I over the three executed experiments. The DCE showed better accuracy while using any of the localization algorithm (UKF, EKF or EH${}_{\infty }$) in both orientation and translation.

**Figure 4:**
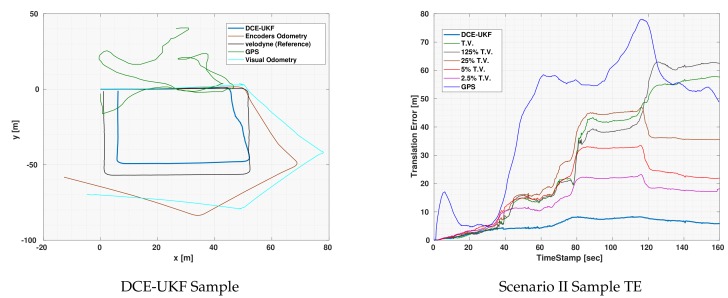
Visual demonstration of one of Scenario II experiments using UKF for localization.

Figure 4 shows the localization output of the DCE-UKF along with the odometries and the GPS measurements. This experiment was executed while the vehicle was moving very close to a building which led to very bad GPS measurements. It can also be seen that both the encoders and the visual odometries suffered from a huge amount of drift. Given all these inaccuracies, through the use of the DCE algorithm and the correction step, the drift in both odometries were eliminated. Notice that although the other runs with constant covariance also had a correction step, the effect of the drift and the inaccuracies in the GPS deteriorated the localization output which shows the efficacy of the DCE algorithm.

Although the GPS error was significantly fluctuating as can be seen in the error plot, the output of the DCE-UKF did not diverge or deteriorate unlike other covariance values. There are two reasons for such results. First, the algorithm relies on calculating the first order polynomial fit of innovations between the odometry and the drift-less sensor, which means that the magnitude of the error in the drift-less sensor itself does not affect the result of the DCE algorithm but only the slope of such error. Second, although the GPS readings suffer from sudden increases in the error, due to the fact that the estimation algorithm does not rely on only one measurement but a series of past measurement (measurement horizon $n_{v}$) to estimate the covariance, the estimated covariance algorithm was accurate enough to get much better results compared to the sensors used and to any other constant covariance.

In Scenario II, the localization output for the TV and 125% diverged which again confirms the fact that using constant covariances for odometries is not accurate as well as finding accurate values for the covariances through experiments is not applicable since the drift behavior changes with operation conditions. Even if the TV can be quantified, using it will not produce accurate localization results and even if it works for a given experiments, it might lead to divergence in other operation conditions or in longer durations of operation.

Table 4, Table 5 and Table 6 shows the quantitative results for scenario II. The DCE algorithm shows better performance than constant covariances for the three used localization algorithms. In the EKF results, the maximum orientation error for other values of covariance was better than that of the DCE algorithm. This is because the experiments for Scenario II was made while the vehicle was moving very close to the walls of a building which might affected the readings of the magnetometer (due to interference with magnetic fields). This led to an underestimation of the magnetometer covariance in this case. Notice that the same constant value for magnetometer covariance was used in all experiments of all scenarios. Again, we stress the fact that even if a set of TV values gave accurate results for a given set of experiments, it might not be the correct value through long operations.

**Figure 5:**
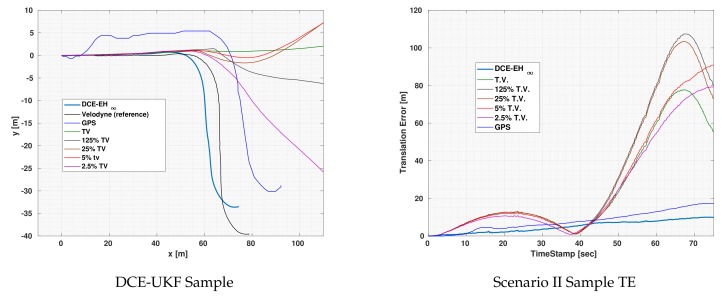
Visual demonstration of one of Scenario II experiments using UKF for localization.

As for Scenario III, Figure 5 shows the path and error plot for one of the experiments using EH${}_{\infty }$ for localization. As shown in the figure, all constant covariance led to the divergence of the localization output. However, in case of DCE, the output did not diverge and shows more accurate results than that of the constant covariances.

In this experiment, the GPS error was suffering from increasing error similar to drift error. However, this drift error was small compared to that of the odometries. Consequently, the error in the localization also suffered from a slight drift. Still, even with this slight drift, the output was significantly better than constant covariances as can be seen in the figure. Notice that the fact that the GPS is suffering from increasing error is not due to the theory of action of the GPS but only a random event that might be due to a certain noise in the path chosen for the vehicle.

Table 7, Table 8 and Table 9 shows the quantitative results for Scenario III which shows superior performance of the DCE algorithm compared to constant covariances. Although the orientation accuracy of some constant covariances were more accurate than the DCE, the value of such covariance differed from one experiment to another and from one filter to another as you might notice in the tables which again leads to the same conclusion that using constant covariance for drift suffering odometries is incomprehensible.

### 6.2. Oxford RobotCar Dataset

Table 10, Table 11 and Table 12 shows the quantitative results for the sessions of Oxford Dataset. As shown in the tables, the covariance estimation algorithm outperformed the constant covariances while using the three fusion filters which shows the efficacy of the algorithm even over longer distances than those tested using the iCab platform and even without any corrective feedback for the odometry being used.

It can be seen that due to the absence of corrective feedback, the average and maximum errors are relatively large. This would not be the case if corrective feedback was incorporated in the results. However, no corrective feedback was made to show the effect of the DCE algorithm over the localization without the effect of the corrections. If corrections were made throughout the runs, the results would have been much better. Despite the fact that the visual odometry will suffer from large error throughout the dataset runs in the absence of the correction due to the accumulation of error with time, the DCE algorithm managed to provide the most accurate results over the dataset runs as shown in the tables.

It is also important to mention specific cases which shows the inconsistency of using constant covariances for odometry fusion. Through using the constant covariance, a given percentage could provide good fusion results (compared to other percentages) for a given experiment but then does not work for another. This can first be seen between the results of different TVs in the results tables. For example, in the Oxford dataset tables, the best UKF results after those of the DCE-UKF is provided by the 2.5% TV constant covariance; however, it is not the case for EKF fusion.

Furthermore, Table 13 shows the best average results through using constant covariance for each of the sessions used from Oxford dataset. The inconsistency of using constant covariance can be obviously seen from the results as in each day, one constant covariance value is better than the others. However, in 3 of the 4 days the DCE algorithm showed superior results compared to all constant covariance values and on average over the whole dataset test, the DCE algorithm was superior using the three fusion filters as shown in Table 10, Table 11 and Table 12.

The reason that in day 4 the DCE was outperformed by the TV is circumstantial because the DCE provides an estimate of the covariance. This does not mean that it provides the optimal or the true value of the covariance but only a good estimate which can be used to get good localization results using odometries which as was shown throughout the paper cannot have a constant covariance and the true value of the covariance cannot be determined at any time or for any kind of operations except retroactively as was done in the results with using a reference. Table 13 is also evidence that the usage of constant covariances is infeasible and will deteriorate the localization results due to the fact that odometries have variable covariance throughout operation.

### 6.3. EU Long-Term Dataset

Table 14 shows the results of the DCE-UKF algorithm compared with different constant covariance similar to the results shown in the previous two subsections. As shown in the table, the results of the DCE-UKF outperform those of the UKF with constant covariances. Those results for the LiDAR odometry (LOAM) conform with the findings of the previous two subsections which shows the results of the DCE algorithm for visual odometry (Oxford Dataset), and visual and encoder odometries (iCab experiments). Although the results of the DCE-UKF compared to constant covariance shows the efficacy of the DCE algorithm, the aim of this subsection and the validation with EU Long-term Dataset is to show the effect of the DCE algorithm when integrated with the LOAM and show the fact that DCE can be integrated with any odometry algorithm in order to estimate its drift covariance and consequently achieve more accurate pose estimation through multisensor fusion.

**Figure 6:**
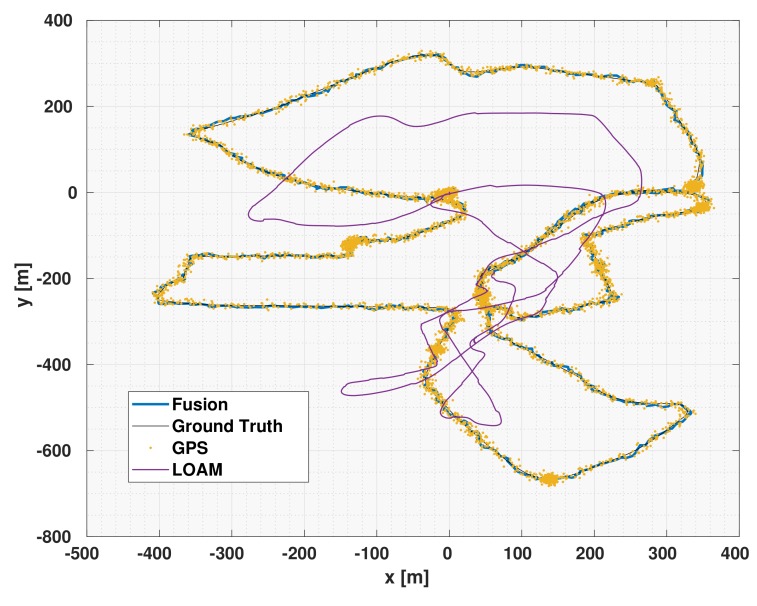
The EU dataset results of the DCE-UKF, LOAM, and GPS.

The data in the EU long-term dataset was recorded while the vehicle was driven with a relatively high speed, consequently, the LOAM algorithm suffered from a substantial amount of drift error. Using the DCE algorithm, we were able to achieve a much better pose estimation. In addition to being better than the LOAM algorithm, we also show here that the DCE results through using the GPS (with added artificial white noise N(0,25)) is better than the pose estimate of the noisy GPS. Figure 6 shows the fusion results of the LOAM with DCE compared to the results of the LOAM alone which suffered from substantial amount of drift as can be shown in the figure. In addition to the LOAM data, the figure also shows the noisy data of the GPS. It can also be seen that the DCE-UKF output is smoother and more accurate than that of the GPS results.

Table 15 shows the $TE_{mean}$ and $TE_{max}$ of the DCE-UKF, LOAM, GPS and filtered GPS over 3 driving sessions of the EU long-term dataset (15,000 km). The use of the DCE-UKF along with the LOAM data achieved much better results than just using the LOAM. This shows the importance of using a fusion algorithm with accurate covariance estimation to achieve more accurate pose estimation than that of an odometry regardless of the amount of drift the odometry might suffer from.

Due to the high amount of drift error in the LOAM results, as shown in Figure 6, one might think that the results of only using a UKF to filter the readings from the GPS might lead to a better results than fusing the GPS with a LiDAR odometry and using the DCE algorithm. For this, we also show the results of filtered GPS readings using a UKF without fusing the results of the LOAM at all. As it can be seen in Table 15, the result of fusing DCE-LOAM with the noisy GPS readings led to better results than ignoring the data from the LOAM completely.

The results achieved from the EU long-term dataset conforms with those of the iCab experiments and the Oxford dataset and show the effect of the DCE algorithm when integrated with a drift suffering odometry. It also confirms the fact that the DCE algorithm can be an integral part of an odometry which can lead to a better pose estimation. The results also show the effect of the DCE algorithm over an even longer driving duration and in different weather conditions.

Finally, all the experiments were executed using an Intel Core i7 CPU at 2.10 GHz processor. The average computation time of the algorithm was 13.3 ms. This small computation time shows the efficiency of using the DCE algorithm on an autonomous vehicle since the performance of the localization or the SLAM modules will not be significantly affected. Furthermore, the maximum and minimum computation time were 23.3 ms and 8.1 ms respectively along with standard deviation of 3.5 ms which shows that the performance of the DCE algorithm is consistent and does not significantly fluctuate through operation.

## 7. Conclusions and Future Work

This paper presented a novel approach for estimating the covariance of odometries which suffers from an accumulation of error (drift) due to the reliance on integration to measure the pose of the vehicle. The algorithm uses the covariance of another sensor which does not suffer from drift (drift-less sensor). The drift covariance estimation algorithm overcomes the challenges of quantifying the constant covariances through the presence of the ground truth or through hard-tuning and also taking into consideration the fact that the covariance of such odometries is dynamic and changes with time during the operation of the sensor used in the odometry depending on different factors.

The drift covariance estimation algorithm was tested using several real-world experiments by using an experimental automated platform as well as Oxford RobotCar Dataset and the EU Long-term Dataset. The testing was done by using three different localization algorithms namely the extended and unscented Kalman filter as well as the extended H${}_{\infty }$ filter. The localization output through using the drift covariance estimation was then compared to the localization output with different constant covariance values. The results showed that the drift covariance estimation algorithm outperformed the constant covariances in almost all the experiments which confirms that the use of constant covariances for drift suffering sensor is neither optimal nor practical. It also shows the efficacy of the drift covariance estimation algorithm from the localization accuracy point of view.

Furthermore, the results of the EU Long-term Dataset were compared with the results of an open source implementation of the well know LOAM algorithm. The results of the comparison showed that integrating the DCE algorithm with the LOAM led to the enhancement of the pose estimation even over longer driving distances and in different weather conditions.

As for the future work, we plan to further investigate possible solutions to generalize the proposed algorithm to work on arbitrary sensors (drift suffering or drift-less).

## Figures and Tables

**Table 1:** Mean of Scenario I UKF results.

Metrics	Mean [%]		Mean [${}^{\circ }$/m]	
	${\mathrm{TE}}_{\mathrm{mean}}$	${\mathrm{TE}}_{\max}$	${\mathrm{OE}}_{\mathrm{mean}}$	${\mathrm{OE}}_{\max}$
DCE-UKF	**1.39**	**3.00**	**0.00281**	**0.1187**
True Variance	6.756	13.853	0.249	0.612
2.5% TV	1.796	3.598	0.077	0.227
5% TV	2.619	5.705	0.099	0.302
25% TV	4.061	7.746	0.164	0.349
125% TV	7.613	14.772	0.263	0.667

**Table 2:** Mean of Scenario I EKF results.

Metrics	Mean [%]		Mean [${}^{\circ }$/m]	
	${\mathrm{TE}}_{\mathrm{mean}}$	${\mathrm{TE}}_{\max}$	${\mathrm{OE}}_{\mathrm{mean}}$	${\mathrm{OE}}_{\max}$
DCE-EKF	**1.591**	**3.267**	**0.026**	**0.09**
True Variance	3.040	7.101	0.058	0.249
2.5% TV	1.655	3.317	0.032	0.099
5% TV	1.717	3.465	0.033	0.109
25% TV	1.954	3.938	0.031	0.105
125% TV	3.811	10.973	0.110	0.374

**Table 3:** Mean of Scenario I EH${}_{\infty }$ results.

Metrics	Mean [%]		Mean [${}^{\circ }$/m]	
	${\mathrm{TE}}_{\mathrm{mean}}$	${\mathrm{TE}}_{\max}$	${\mathrm{OE}}_{\mathrm{mean}}$	${\mathrm{OE}}_{\max}$
DCE-EH${}_{\infty }$	**2.289**	**4.116**	**0.031**	**0.091**
True Variance	20.850	56.460	0.474	1.580
2.5% TV	2.434	4.416	0.040	0.137
5% TV	2.510	4.575	0.037	0.135
25% TV	7.183	15.346	0.138	0.372
125% TV	19.247	45.620	0.351	1.189

**Table 4:** Mean of Scenario II UKF results.

Metrics	Mean [%]		Mean [${}^{\circ }$/m]	
	${\mathrm{TE}}_{\mathrm{mean}}$	${\mathrm{TE}}_{\max}$	${\mathrm{OE}}_{\mathrm{mean}}$	${\mathrm{OE}}_{\max}$
DCE-UKF	**2.00**	**3.39**	**0.019**	**0.0720**
True Variance	13.034	44.662	0.019	0.109
2.5% TV	4.942	8.259	0.019	0.111
5% TV	6.498	11.451	0.019	0.103
25% TV	9.633	21.433	0.020	0.111
125% TV	13.593	60.060	0.019	0.110

**Table 5:** Mean of Scenario II EKF results.

Metrics	Mean [%]		Mean [${}^{\circ }$/m]	
	${\mathrm{TE}}_{\mathrm{mean}}$	${\mathrm{TE}}_{\max}$	${\mathrm{OE}}_{\mathrm{mean}}$	${\mathrm{OE}}_{\max}$
DCE-EKF	**2.539**	**4.474**	**0.019**	0.097
True Variance	10.283	28.131	0.020	0.079
2.5% TV	3.852	6.663	0.024	0.089
5% TV	4.310	7.735	0.020	**0.074**
25% TV	8.057	23.822	0.020	0.077
125% TV	9.813	24.6910	0.021	0.075

**Table 6:** Mean of Scenario II EH${}_{\infty }$ results.

Metrics	Mean [%]		Mean [${}^{\circ }$/m]	
	${\mathrm{TE}}_{\mathrm{mean}}$	${\mathrm{TE}}_{\max}$	${\mathrm{OE}}_{\mathrm{mean}}$	${\mathrm{OE}}_{\max}$
DCE-EH${}_{\infty }$	**3.585**	**5.964**	**0.023**	**0.126**
True Variance	47.930	122.489	0.268	1.096
2.5% TV	15.928	32.895	0.250	0.871
5% TV	35.600	67.563	0.505	1.167
25% TV	45.915	101.055	0.329	1.083
125% TV	47.359	134.881	0.262	1.123

**Table 7:** Mean of Scenario III UKF results.

Metrics	Mean [%]		Mean [${}^{\circ }$/m]	
	${\mathrm{TE}}_{\mathrm{mean}}$	${\mathrm{TE}}_{\max}$	${\mathrm{OE}}_{\mathrm{mean}}$	${\mathrm{OE}}_{\max}$
DCE-UKF	**2.11**	**4.23**	0.0251	**0.0763**
True Variance	15.841	56.230	**0.022**	0.167
2.5% TV	4.375	8.971	0.023	0.090
5% TV	4.613	10.372	0.024	0.120
25% TV	7.627	21.715	0.025	0.147
125% TV	13.508	46.674	0.024	0.155

**Table 8:** Mean of Scenario III EKF results.

Metrics	Mean [%]		Mean [${}^{\circ }$/m]	
	${\mathrm{TE}}_{\mathrm{mean}}$	${\mathrm{TE}}_{\max}$	${\mathrm{OE}}_{\mathrm{mean}}$	${\mathrm{OE}}_{\max}$
DCE-EKF	**2.225**	**5.804**	0.044	0.153
True Variance	3.116	7.826	0.025	0.142
2.5% TV	3.215	8.614	0.028	0.168
5% TV	3.389	8.009	0.030	0.154
25% TV	3.063	7.740	0.027	**0.122**
125% TV	3.028	8.194	**0.024**	0.149

**Table 9:** Mean of Scenario III EH${}_{\infty }$ results.

Metrics	Mean [%]		Mean [${}^{\circ }$/m]	
	${\mathrm{TE}}_{\mathrm{mean}}$	${\mathrm{TE}}_{\max}$	${\mathrm{OE}}_{\mathrm{mean}}$	${\mathrm{OE}}_{\max}$
DCE-EH${}_{\infty }$	**4.624**	**9.393**	**0.037**	**0.153**
True Variance	27.546	81.960	0.375	1.739
2.5% TV	20.756	61.027	0.318	1.230
5% TV	26.518	80.397	0.344	1.343
25% TV	27.829	85.320	0.399	1.790
125% TV	25.990	79.923	0.291	1.467

**Table 10:** Mean of Oxford Dataset UKF results.

Metrics	Mean [%]	
	${\mathrm{TE}}_{\mathrm{mean}}$	${\mathrm{TE}}_{\max}$
DCE-UKF	**2.744**	**9.1085**
True Variance	3.5871	14.1377
2.5% TV	2.9323	9.194
5% TV	3.3549	10.7285
25% TV	4.3890	36.8538
125% TV	3.5632	13.6774

**Table 11:** Mean of Oxford Dataset EKF results.

Metrics	Mean [%]	
	${\mathrm{TE}}_{\mathrm{mean}}$	${\mathrm{TE}}_{\max}$
DCE-EKF	**3.2265**	**10.0566**
True Variance	3.6925	14.1595
2.5% TV	3.3623	11.7762
5% TV	3.2507	11.2075
25% TV	4.2876	35.1085
125% TV	3.7339	14.3134

**Table 12:** Mean of Oxford Dataset EH${}_{\infty }$ results.

Metrics	Mean [%]	
	${\mathrm{TE}}_{\mathrm{mean}}$	${\mathrm{TE}}_{\max}$
DCE-EH${}_{\infty }$	**8.9024**	**29.9001**
True Variance	23.5772	60.7318
2.5% TV	17.6122	44.6157
5% TV	20.1490	50.1683
25% TV	24.2690	64.7884
125% TV	24.5318	62.4231

**Table 13:** The best average UKF error using constant covariances for each day of the dataset and the corresponding DCE-UKF results.

Day Number	TE${}_{\mathrm{mean}}$ [%]	
	Best Constant Covariance	Adaptive Covariance
1	1.509 (2.5 % T.V.)	**1.366**
2	2.886 (125 % T.V.)	**2.584**
3	5.759 (5 % T.V.)	**5.295**
4	**2.984** ( T.V.)	3.471

**Table 14:** Mean of EU Dataset UKF results.

Metrics	Mean [%]	
	${\mathrm{TE}}_{\mathrm{mean}}$	${\mathrm{TE}}_{\max}$
DCE-UKF	**0.078**	**0.295**
True Variance	0.679	2.280
2.5% TV	0.081	0.314
5% TV	0.088	0.469
25% TV	0.867	2.130
125% TV	0.096	1.364

**Table 15:** The EU dataset results of the DCE-UKF, LOAM, GPS and filtered GPS.

Metrics	Mean [%]	
	${\mathrm{TE}}_{\mathrm{mean}}$	${\mathrm{TE}}_{\max}$
DCE-UKF	**0.078**	**0.295**
LOAM	6.155	13.607
GPS	0.106	0.380
Filtered GPS	0.082	0.319
