# Correction: Osman, M., et al. A Novel Online Approach for Drift Covariance Estimation of Odometries Used in Intelligent Vehicle Localization. *Sensors* 2019, *19*, 5178

**DOI:** 10.3390/s20041162

**Published:** 2020-02-20

**Authors:** Mostafa Osman, Ahmed Hussein, Abdulla Al-Kaff, Fernando Garcia, Dongpu Cao

**Affiliations:** 1 Mechanical and Mechatronics Engineering, University of Waterloo, Waterloo, ON N2L 3G1, Canada; 2 Intelligent Driving Function Department, IAV GmbH, 10587 Berlin, Germany; ahmed.hussein@ieee.org; 3 Intelligent Systems Lab (LSI), Universidad Carlos III de Madrid (UC3M), 28911 Leganes, Spain; akaff@ing.uc3m.es (A.A.-K.); fegarcia@ing.uc3m.es (F.G.); 4 Waterloo Cognitive Autonomous Driving (CogDrive) Lab, University of Waterloo, Waterloo, ON N2L 3G1, Canada; dongpy.cao@uwaterloo.ca

The authors wish to make the following corrections to this paper [1]:

On page 6 of the paper [1], the calculation of the drift increment covariance has an error, where the transpose should be on the second bracket instead of the first. The equation should be:

$$Q_{\delta }=E\left[\left(K_{1}\Delta z-\mu_{\delta }\right){\left(K_{1}\Delta z-\mu_{\delta }\right)}^{T}\right]\;=E\left[K_{1}\Delta z\Delta z^{T}K_{1}-2\mu_{\delta }K_{1}\Delta z^{T}+\mu_{\delta }\mu_{\delta }^{T}\right]\;=E\left[K_{1}\Delta z\Delta z^{T}K_{1}\right]-\mu_{\delta }\mu_{\delta }^{T}$$

This error propagated to several equations in the paper, as follows:

Equation (16) on page 6 of the paper [1], will be:

$$Q_{\delta_{k}}=E\left[K_{1}\Delta z\Delta z^{T}K_{1}\right]$$

and Equation (17) becomes:

$$\sum_{\delta_{k}}=\sum_{\delta_{k-1}}+Q_{\delta_{k}}$$

Equation (26) changes to:

$${\hat{Q}}_{\delta_{k}}=E[K_{1}\Delta {\hat{z}}_{\delta_{k}}\Delta {\hat{z}}_{\delta_{k}}^{T}K_{1}]\frac{1}{q}\overset{q}{\underset{i=0}{\sum }}{\hat{K}}_{i}\Delta {\hat{z}}_{\delta_{k}}\Delta {\hat{z}}_{\delta_{k}}^{T}{\hat{K}}_{i}$$

Equation (27) in paper [1] is removed, Equation (28) in paper [1] becomes Equation (27) and, finally, Equation (30) is changed to:

$${\hat{Q}}_{\delta_{k}}=\overset{2m}{\underset{i=0}{\sum }}W_{i}^{\left(m\right)}{\hat{K}}_{i}\Delta {\hat{z}}_{\delta_{k}}\Delta {\hat{z}}_{\delta_{k}}^{T}{\hat{K}}_{i}$$

Note that the two equations were corrected as follows in step 8 of Algorithm 1:

$${\hat{Q}}_{\delta_{k}}=\frac{1}{q}\overset{q}{\underset{i=0}{\sum }}{\hat{K}}_{i}\Delta {\hat{z}}_{\delta_{k}}\Delta {\hat{z}}_{\delta_{k}}^{T}{\hat{K}}_{i}$$

$${\hat{\sum }}_{\delta_{k}}={\hat{\sum }}_{\delta_{k-1}}+W{\hat{Q}}_{\delta_{k}}$$

The authors would like to apologize for any inconvenience caused to the readers by these changes and would like to mention that the algorithm was developed and tested properly and the mentioned error in the paper is due to a typo.
